# Concurrent Analysis of Tiafenacil and Its Transformation Products in Soil by Using Newly Developed UHPLC-QTOF-MS/MS-Based Approaches

**DOI:** 10.3390/ijms25158367

**Published:** 2024-07-31

**Authors:** Wenwen Zhou, Anqi Yan, Shujie Zhang, Dayong Peng, Jun Li

**Affiliations:** 1 College of Food Science and Engineering, Jiangxi Agricultural University, Nanchang 330045, China; zhangsj@163.com; 2 School of Agriculture, Food and Ecosystem Sciences, University of Melbourne, Parkville, VIC 3052, Australia; anqi.yan1@student.unimelb.edu.au; 3 College of Chemistry and Materials, Jiangxi Agricultural University, Nanchang 330045, China; dayongpeng@163.com

**Keywords:** tiafenacil, dissipation kinetics, transformation products, QuEChERS, UHPLC-QTOF-MS/MS, density functional theory (DFT)

## Abstract

As new pesticides continue to emerge in agricultural systems, understanding their environmental behavior is crucial for effective risk assessment. Tiafenacil (TFA), a promising novel pyrimidinedione herbicide, was the focus of this study. We developed an efficient QuEChERS-UHPLC-QTOF-MS/MS method to measure TFA and its transformation products (TP1, TP2, TP3, TP4, and TP5) in soil. Our calibration curves exhibited strong linearity (R^2^ ≥ 0.9949) ranging from 0.015 to 2.0 mg/kg within a low limit of quantification (LOQ) of 2.0 µg/kg. Inter-day and intra-day recoveries (0.10 to 2.0 mg/kg, 80.59% to 110.05%, RSD from 0.28% to 12.93%) demonstrated high sensitivity and accuracy. Additionally, TFA dissipation under aerobic conditions followed first-order kinetics, mainly yielding TP1 and TP4. In contrast, TP1 and TP2 were mainly found under sterilized and anaerobic conditions, and TFA dissipation followed second-order kinetics. Moreover, we predicted the transformation pathways of TFA using density functional theory (DFT) and assessed the toxicity levels of TFA and its TPs to aquatic organisms using ECOSAR. Collectively, these findings hold significant implications for a better understanding of TFA fate in diversified soil, benefiting its risk assessment and rational utilization.

## 1. Introduction

The environmental risk posed by parent pesticides has become a widespread concern. When developing new pesticides, their transformation behavior and ecotoxicological effects in the environment should be considered by assessing their bio-risk before admitting them to public [1]. Surprisingly, numerous studies have revealed that 51% of transformation products (TPs) exhibit greater toxicity than the directly utilized pesticides. Furthermore, 10-fold toxicity in 9% of TPs was characterized, compared to their parental chemicals, potentially challenging environmental ecosystems and human health [2]. Regarding this, the transformation and kinetics mechanisms related to these newly developed pesticides and their transformation products need clarified.

The dissipation process of pesticides in soil is highly complicated, and their dissipation rates are influenced by a combinatorial set of soil physicochemical properties (such as soil type, soil pH, soil moisture content) and environmental factors (such as initial concentration, presence of microorganisms). For example, the dissipation rate constants (k) for a variety of pesticides (such as glyphosate, 2,4-D, chlorothalonil, and dimethoate) are inversely proportional to soil organic carbon (OC) and clay content, in contrast to being directly proportional to the pH and sand content. Interestingly, the calculated half-life values (t_1/2_) of the aforementioned four pesticides are positively correlated with the OC and clay content and negatively correlated with the soil pH and sand content [3]. The dissipation of seven neonicotinoids (clothianidin, thiamethoxam, imidacloprid, acetamiprid, dinotefuran, thiacloprid, and nitenpyram) in soil follows first-order kinetics, with t_1/2_ ranging from 33 to 305 days. The soil moisture content, clay content, and cation exchange capacity improved the dissipation rate of neonicotinoid insecticides [4]. Therefore, by determining the k and t_1/2_ of pesticides, their risks to the environment and human health can be assessed [5].

Radioisotope labeling and quadrupole mass spectrometry are pivotal techniques for investigating chemical dissipation pathways and identifying metabolites [6]. However, the high cost of isotope-labeled compounds limits metabolite identification. From this scenario, the development of more advanced techniques to streamline the dissipation pathway is urgently required [7]. In the context of accurate identification of chemicals and their intermediates, a series of pre-treatment and detection approaches based on ultra-high-performance liquid chromatography–quadrupole time-of-flight mass spectrometry (UHPLC-QTOF-MS)-based represents a notable breakthrough [8]. Both UHPLC and HPLC achieve improved resolution through the use of shorter chromatography columns and reduced working times, enabled by the detection of smaller particle sizes and the application of higher pressures. TOF or QTOF instruments offer superior accuracy and resolution in metabolite characterization, alongside a simple identification process [9].

Tiafenacil (TFA) is a new urea-pyrimidine-based herbicide developed in 2012 by Foo Ahmu Hanong in Korea, of which the core structure consists of amide and pyrimidinedione with a β-amino acid backbone as a protoporphyrinogen oxidase (PPO) inhibitor (Figure S1) [10]. Both 5% TFA suspension and 70% TFA aqueous dispersion granules could effectively control grassy and broad-leaved weeds in citrus orchards, especially prominent activity against glyphosate-resistant weeds such as *Eleusine indica* (L.) Gaertn and *Erigeron canadensis* L. [11]. Together, TFA has excellent potential to replace glyphosate for weed control on citrus orchards, corn, cotton, soybeans, wheat, grapes, fallow, and non-cropped areas [12]. In addition, worldwide studies on TFA mainly focus on its residue analysis [11], synthetic routes [13], herbicidal activity [14], and damage to zebrafish [15]. In other words, transformation behaviors of TFA in the context of actual scenarios have not been identified. However, the investigation on TFA’s transformation and diverse derivatives provides further insights into its environmental behavior and dynamics [16]. Furthermore, a better understanding of the toxicity of both TFA mitigation and its metabolites is critical for comprehensive risk assessment to enable effective monitoring and safeguarded biosafety.

Together, we aimed to achieve multiple objectives: to (1) establish an extraction and analysis method for TFA and its metabolites, (2) conduct a laboratory investigation on TFA dissipation and diversified metabolites across four soil types, (3) perform theoretical model calculations on degrading chemicals and utilize the ECOSAR program to assess the toxicity of TFA and its metabolites to aquatic organisms, and (4) propose likely pathways of TFA dissipation in soil. Notably, we attempted to develop a novel quantitative approach to detect TFA and its metabolites in soil, by using UHPLC-QTOF-MS/MS-embedded pre-treatment and parameters. This advancement would significantly benefit our understanding of TFA’s environmental characteristics and practical implications.

## 2. Results and Discussion

### 2.1. Optimization of Instrument Conditions

#### 2.1.1. MS/MS Parameters

**Figure 1:**
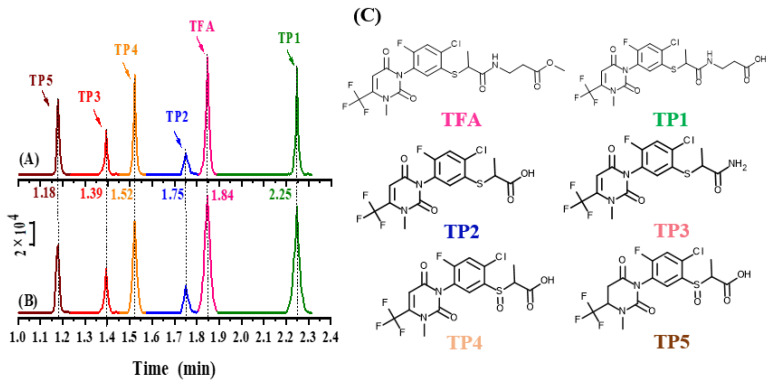
Typical chromatograms for chemicals under study. (**A**) Standard chemicals, (**B**) spiked samples and (**C**) chemical structures of TFA and its TPs. The spiked concentration of TFA and its TPs in soil for each compound was indicated, respectively.

TFA and a total of five metabolites were detected by using the MRM mode in MS/MS. As a result, all six target compounds were ionized more effectively in the positive ion mode than in the negative ion mode. The precursor ion [M + H]^+^ was selected for each analyte in the positive ESI mode. The abundance of the precursor ion for each target compound was determined by optimizing the fragment voltage in the selected ion monitoring mode. The precursor ions [M + H]^+^ were further decomposed at varying collision energies. According to EU guidance document SANTE/11312/2021, it is necessary to analyze most compounds based on two ion transitions to accurately identify compounds arising from the four identification points (IPs). Each analyte was quantified and identified using the two fragment ions with the highest abundance. The less abundant ion transition was used for identification, while the more abundant ion transition was used for quantification [17]. Molecular weight, precursor ions, fragment voltages, and corresponding collision energies are provided in Table 1 and Figure 1.

#### 2.1.2. Extraction and Clean-Up Pretreatment

We tested various sorbents and extraction solvents to extract and purify TFA and its five metabolites effectively. To determine the best extraction and purification methods for the target compounds, we conducted a recovery test and analyzed the recovery values of the samples.

**Figure 2:**
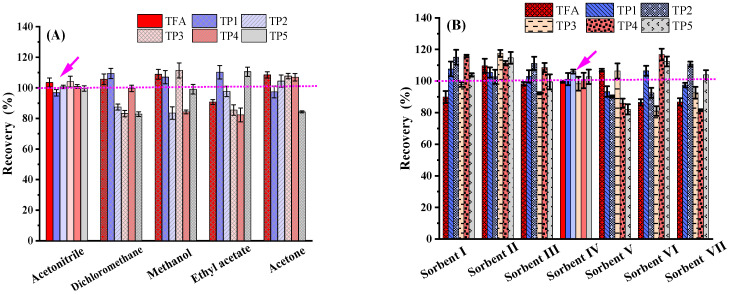
The detection recovery by using different solvents and sorbents. (**A**) Solvent: acetonitrile, dichloromethane, methanol, ethyl acetate, and acetone); (**B**) sorbent: I: 50 mg C18 + 150 mg MgSO_4_, II: 50 mg PSA + 150 mg MgSO_4_, III: 5 mg GCB + 150 mg MgSO_4_, IV: 5 mg MWCNT + 150 mg MgSO_4_, V: 50 mg C18 + 50 mg PSA + 150 mg MgSO_4_, VI: 50 mg C18 + 5 mg GCB + 150 mg MgSO_4_, VII: 50 mg C18 + 50 mg PSA + 5 mg GCB + 150 mg MgSO_4_. Of note, TFA and its TPs in ferralsol matrix were tested at the 100 µg/kg level in acetonitrile (n = 3).

To achieve satisfactory recoveries, multi-residue analysis requires complete extraction of the target compound present in the specimen. To explore this, powerful extraction solvents such as acetonitrile, dichloromethane, methanol, ethyl acetate, and acetone were evaluated at a 100 μg/kg spiked level, following the quick, easy, cheap, effective, rugged, and safe (QuEChERS) extraction concept [18]. Recovery tests for all target compounds demonstrated 81.02 to 116.78% herbicide recoveries (as shown in Figure 2A). Acetonitrile was ultimately chosen as the extraction solvent for further studies, as it effectively minimized the solubilization of polar compounds.

To obtain unadulterated samples with minimal interference, a study was conducted on four commonly used adsorbents—primary secondary amine (PSA), graphitized carbon black (GCB), octadecylsilane (C18), and multi-walled carbon nanotubes (MWCNTs). The study, conducted in acetonitrile, aimed to determine their efficacy in removing impurities from selected matrices. Each adsorbent exhibited a different purification mechanism, as depicted in Figure 2B. PSA functions via weak anion exchange and is particularly effective in eliminating matrix compounds like fatty acids and sugars [19]. C18 is well-suited for isolating chemicals with non-polarity or moderate polarity. GCB operates through hydrophobic interactions and is best suited for removing compounds such as chlorophylls and carotenoids. MWCNTs, due to their elongated tube morphologies, porous surfaces, and large specific surface areas, are highly effective in absorbing pigment-based impurities. It is this unique feature that accounts for the exceptional purification effect of MWCNTs when dealing with complex matrices [20]. Anhydrous MgSO_4_ removes water from the sample, improving extraction efficiency and helping to reduce interference in subsequent analyses such as peak broadening or tailing [21]. We chose these adsorbents to remove interfering substances in the soil, such as organic matter, pigments, and proteins. These impurities can affect the measurement of compounds, hence the adoption of the QuEChERS method. This method is widely used in many matrix measurements to ensure the accuracy of the analysis. For example, it has been applied in the analysis of propyrisulfuron in natural paddy field environments using QuEChERS@UPLC-Q-TOF-MS/MS [22]. This approach also facilitates the co-determination of tiafenacil and its six metabolites in fruits [11], as well as the analysis of multiple antibiotics in the soil–rice system [21].

Seven sorbent combinations were tested, considering the soil’s various organic acids and pigments. As shown in Figure 2B, the results indicate that when C18 was utilized for soil matrix clean-up, TFA, TP4, and TP5 recoveries were relatively low. The result difference is that C18 adsorbs not only pigments and fatty acids but also these compounds, leading to lower recoveries. We opted for sorbent IV in this study to account for the high levels of organic acids, pigments, and fatty acids in the soils. The EU SANTE/11312/2021 states that the quantitative analysis related to remaining pesticide in soil should yield a recovery between 70% and 120% with an RSD of ≤20% [17]. Using adsorbent IV (5 mg MWCNT + 150 mg MgSO_4_) resulted in a satisfactory recovery of TFA and its TPs.

### 2.2. Method Performance

#### 2.2.1. Specificity, Linearity, LOQ, and ME

We have evaluated the method’s specificity and found no interference during the retention time of the intended compound. Table 2 summarizes the linearity, LOQ, and ME. The results show that good linearities were achieved for all target compounds across all matrices, with excellent correlation coefficients (R^2^ ≥ 0.9949, 15~2000 μg/kg). The LOQs for TFA and its five metabolites were estimated to be 2.0 µg/kg below the MRLs of 10 µg/kg specified in US MRL for agricultural soils [23]. It appears that our assay for detecting TFA and its metabolites has a lower LOQ value compared to the published method in fruit [11].

#### 2.2.2. Precision, Accuracy, and Measurement Uncertainty

We evaluated the accuracy and precision of our proposed method for measuring the recovery of TFA and its metabolites in soil matrices with a relative standard deviation (RSD). We conducted this assessment over three days, using five replicates at three different concentration levels (0.1, 0.5, and 2.0 mg/kg). The mean recovery values ranged from 80.59% to 110.05%, indicating the method’s precision. Additionally, we tested the reproducibility for all matrices and target compounds, with the intra-day (n = 5) RSD_r_ and inter-day (n = 5) RSD_R_ ranging from 0.28% to 11.55% and 0.29% to 12.93%, respectively (Table S1). Measurement uncertainty calculation is an essential parameter for the metrological evaluation of results. The U(y) values for TFA and five transformation products were below 23.7% and 38.8%, respectively. The contributions of six uncertainty factors to Uc(y) are presented in Figure S2. The relative uncertainties from calibration curves (Uc) and recovery (Um) were the most significant factors in all four matrices, contributing approximately 40% and 30% to Uc(y), respectively. Additionally, according to the SANTE/12682/2021 report, the U(y) values of all analytes in the four matrices were within acceptable ranges: U(y) was less than 40% [24]. Based on the European Union Guidance Document [17], our developed method based on UHPLC-QTOF-MS/MS for detecting herbicide residue has provided accurate and precise results for analyzing TFA and its soil metabolites.

#### 2.2.3. Comparison of Established Methods

For this method, the application scope for simultaneous determination of TFA and its five transformation products as target compounds has been extended to soil matrices to accurately assess risks and ensure environmental safety. Moreover, the analysis time is only 2.25 min, which is shorter than previously reported times for TFA in soil matrices. Many laboratory-validated analytical methods have been published. These methods measure TFA and six metabolites in fruits (apple, orange, grape, mango, banana, pear and peach) using ultra-high-performance liquid chromatography/tandem mass spectrometry (UHPLC-MS), with retention times for TFA and its metabolites close to 3 min [11]. Gao established a high-performance liquid chromatography–mass spectrometry (HPLC-MS) method to determine TFA in soil, using a modified QuEChERS focusing on five types of soil samples, and determining TFA within 5 min [19]. The analysis time is more than double that of our study. The LOQs, defined as the lowest point of the spiked levels test, for six analytes in various matrices are all 2 µg/kg (Table 3), which are lower than the maximum residue limits (MRLs). Most importantly, the method in this study exhibits lower LOQs than other methods, along with a lower relative standard deviation value and a wider linear range.

### 2.3. Dissipation Dynamics of TFA in Soil

The dissipation of TFA in diverse soil samples was assessed using both first- and second-order kinetic models, revealed to be significant (*p* ≤ 0.01). The second-order model excelled in predicting TFA dissipation in aerobic conditions across all soils (Table S2), while the first-order model performed better in sterilized and anaerobic conditions. However, the first-order model tended to overestimate TFA dissipation in aerobic conditions (Figure S3), yielding higher t_1/2_ values compared to the second-order model (Anthrosols = 13.00 h vs. 10.63 h; Ferralsols = 4.15 h vs. 4.06 h; Lixisols = 5.27 h vs. 3.38 h; Gleysols = 5.00 h vs. 3.71 h). Conversely, in sterilized and anaerobic conditions, the second-order model tended to underestimate dissipation rates, resulting in lower t_1/2_ values compared to the first-order model (Anthrosols = 23.26 h vs. 18.68 h; Ferralsols = 15.43 h vs. 11.83 h; Lixisols = 18.48 h vs. 13.40 h; and Gleysols = 14.38 h vs. 11.44 h under sterilized conditions and Anthrosols = 15.89 d vs. 12.12 d; Ferralsols = 3.05 d vs. 3.52 d; Lixisols = 5.75 d vs. 4.58 d; and Gleysols = 4.60 d vs. 2.89 d under anaerobic conditions. These findings underscore the importance of selecting models based on the underlying dissipation pattern and their alignment with the observed data.

Based on testing conducted under aerobic and anaerobic conditions, it was observed that TFA has a rapid dissipation rate (t_1/2_ ≤ 30 d). Testing was performed according to the guidelines outlined in NY/T 3150-2017 “Guidelines for evaluating and calculating dissipation kinetics in environmental media for pesticide registration” [25] to examine the transformations occurring in soil.

The aerobic dissipation rate is approximately 3.5 and 24 times higher than the sterilized and anaerobic dissipation rates, respectively. This observation leads to the conclusion that TFA transformation is predominantly influenced by aerobic dissipation. Aerobic microorganisms are likely predominant to TFA dissipation or its acceleration. In low light conditions, herbicide breakdown in the soil is mainly caused by microbial and abiotic dissipation [26]. For example, the dissipation rates of 2,4,4′-tribromodiphenyl ether (BDE 28), decabromodiphenyl ether (BDE 209), tetrabromobisphenol A (TBBPA), 1,2-dibromo-4-(1,2-dibromoethyl) cyclohexane (TBECH), 2,4,6-tribromophenol (246BrPh), and hexabromobenzene (HxBrBz) were much higher under aerobic than those in anaerobic conditions, due to their shared halogenated aromatic structures [27].

**Figure 3:**
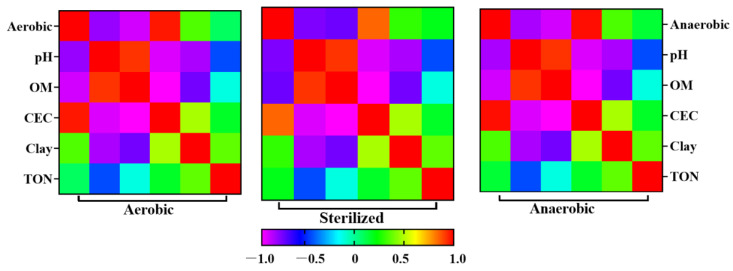
The relationship between the half-life of TFA in soil and its underlying physicochemical properties examined through the application of the Pearson correlation method under aerobic, sterilized and anaerobic conditions.

We performed a multiple linear regression analysis to explore the relationship between TFA’s half-life and soil physicochemical parameters in both aerobic and anaerobic conditions. Figure 3 and Table S3 clearly depict a significant positive correlation between pH and OM (organic matter) within the half-life, with a noteworthy positive correlation with the CEC (cation exchange capacity). Moreover, in conjunction with the DFT computational outcomes, it is evident that microbes may have a pivotal role in the conversion of TFA into TP1 and TP2. Consequently, soil pH directly impacts microbial activity and the chemical composition of the ecosystem [28]. As a result, an acidic pH tends to decelerate the dissipation rate, resulting in prolonged half-lives. Soil OM contributes to the immobilization of organic chemicals in the soil, and the richness and activity of the soil microbiome are closely associated with the OM content [29]. In addition, OM plays a crucial role in the dissipation of chemicals in soil by acting as an extracellular electron acceptor and facilitating electron transfer reduction [30]. CEC mirrors the soil particles’ ability to retain and exchange ions. Soils with higher CEC typically possess surfaces with stronger negative charges, facilitating the robust adsorption of positive ions like calcium, magnesium, and potassium [31]. This can significantly influence the adsorption and release of pesticide molecules in the soil, thereby affecting their dissipation rate [32]. The cumulative effects of these factors determine the fate of pesticides within soil. Discrepancies in soil types, pesticide properties, and environmental conditions can lead to variations in pesticide dissipation processes. Therefore, a holistic consideration of these elements proves imperative in practical applications to enhance the prediction and management of pesticide behavior in soil.

**Figure 4:**
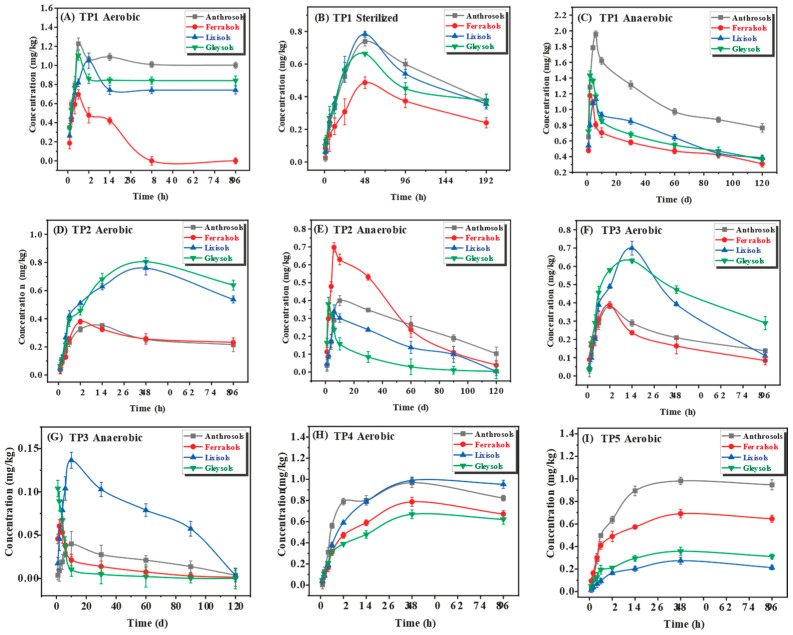
The residue variation of TPs under diverse conditions. (**A**) TP1 under aerobic conditions, (**B**) TP1 under sterilized conditions, (**C**) TP1 under anaerobic conditions, (**D**) TP2 under aerobic conditions, (**E**) TP2 under anaerobic conditions, (**F**) TP3 under aerobic conditions, (**G**) TP3 under anaerobic conditions, (**H**) TP4 under aerobic conditions, and (**I**) TP5 under aerobic conditions.

Figure 4 shows the detection of all five transformation products under aerobic conditions across four distinct soils. However, under sterilized conditions, only TP1 was identified, with TP4 and TP5 absent under anaerobic conditions. Notably, in aerobic settings, TP1 and TP4 emerged as the primary transformation products, peaking at 6 and 48 h, respectively. Conversely, TP2, TP3, and TP5 peaked within 12 to 48 h. Under sterilized conditions, TP1 peaked at 48 h, while under anaerobic conditions, TP1 and TP2 were the dominant transformation products, peaking at 4 and 6 days, respectively. TP3 peaked within 1 to 10 days and dissipated subsequently.

Lastly, we analyzed samples of Lixisols and Gleysols to assess the accuracy of the trained pipeline for detecting trace levels of TFA and its metabolites. After following the sample preparation procedure outlined in Section 3.1, we analyzed the samples using UHPLC-QTOF-MS/MS. Our findings revealed that the concentrations of TFA and its metabolites in all tested samples were below LOQ.

### 2.4. DFT-Based Prediction of TFA Transformants

TFA’s molecular properties were assessed using DFT to examine potential dissipation reactions. In Figure S1, identified reactive sites exposed the vulnerability of trifloxysulfuron’s pyrimidinedione structure to ring breakage when exposed to nucleophilic reagents such as -OH.

**Figure 5:**
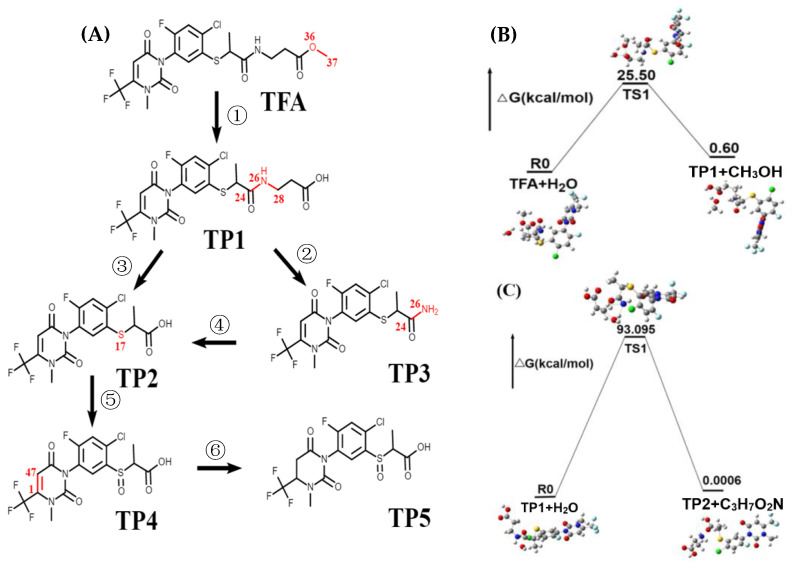
Proposed dissipation pathways of TFA. (**A**) Six mainly characterized chemical reactions: ① ester cleavage, ② amide hydrolysis, ③ amide cleavage, ④ amide hydrolysis, ⑤ sulfur oxidation, and ⑥ nitrogen heterocyclic reduction. Mechanistic energy diagrams of the reaction, at the B3LYP/6–311G(d) level: (**B**) TFA + H_2_O → TP1 + CH_3_OH, (**C**) TP1 + H_2_O → TP2 + C_3_H_7_O_2_N.

In order to determine the best approach when using nucleophilic or electrophilic reagents, Table S4 provides information on the natural charge, bond length and its order in TFA. It is recommended that target atoms possess high natural charge depletion. For TFA, the carbon bond most susceptible to nucleophilic or electrophilic attack is shown in Table S5. Chemical bonds break more efficiently when the bond order is smaller, and breaking longer bonds is easier [33]. Hence, amides and pyridine di-ones are the primary reactive sites. Radical attack on the amides and pyridine di-ones bond of TFA can result in six reactions (Figure 5A): (I) the ester bond O(36)-C(37) in TFA breaks to form TP1; (II) the amide bond C(24)-N(26) in TP1 breaks to form TP2; (III) C(24)-N(26) fracture in TP1 to form TP3; (IV) TP3 undergoes amide bond C(24)-N(26) breakage to form TP2; (V) sulfur oxidation of TP2 occurs to form TP4; (VI) C(1)-C(47) in TP4 undergoes a further reducing reaction to form TP5. In our analysis, H_2_O is an initial reactant in dissipation reactions. We proposed reaction mechanisms: TFA + H_2_O → TP1 + CH_3_OH (B), TP1 + H_2_O → TP2 + C_3_H_7_O_2_N (C) (Figure 5B,C display schematic diagrams of each step, respectively).

Both reactions share the transformation pattern triggered by the oxygen atom of H_2_O to attack the electron-deficient atom, resulting in two-membered transition states (TSs). The energy barrier of the reaction from TP1 to TP2 is too high for this reaction to occur under natural conditions. In most cases, the reaction cannot happen spontaneously if the energy barrier is higher than 25 kcal/mol [34]. We speculate that are reaction processes involving microorganisms.

### 2.5. Risk Assessment

Numerous studies have utilized the ECOSAR technique to evaluate the impact of different pesticide compounds and their metabolites on aquatic organisms [35]. This is accomplished by predicting the acute and chronic toxicity of fish, daphnia, and green algae.

**Figure 6:**
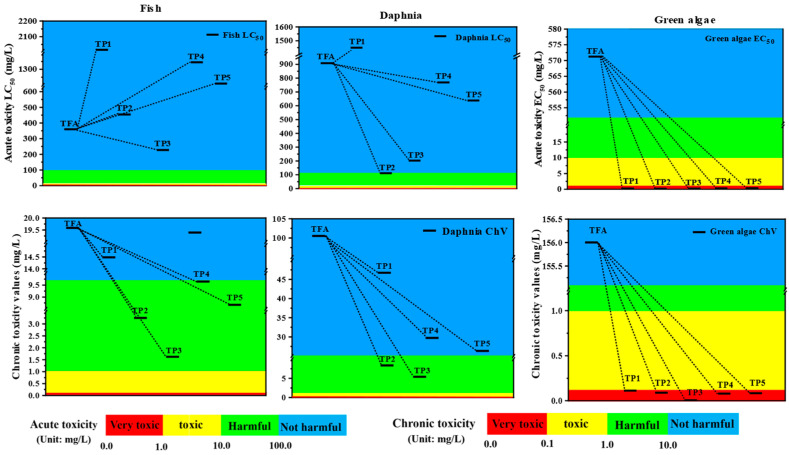
Acute and chronic toxicities for TFA and its metabolites (unit: mg/L). Blue, green, yellow, and red rectangles indicate the toxicity properties of studied compounds, respectively.

The toxicity levels of TFA and its TPs towards fish, daphnia, and green algae are illustrated in Figure 6 and Table S6. Based on the criteria established by the European Union and China, the toxicity levels of the examined species are categorized. The findings indicate that TFA posed no harm to fish, Daphnia, and green algae, exhibiting acute toxicity values of 352.000, 905.000, and 571.000 mg/L and chronic toxicities of 19.600, 101.000, and 156.000 mg/L, respectively. Therefore, we confirmed that TFA is a non-toxic substance for the studied organisms.

Likewise, the transformants showed no harm to fish and Daphnia, displaying acute toxicity LC_50_ within the range of 226.186~2016.184 mg/L and 113.899~1451.282 mg/L, respectively. However, the transformants exhibited high toxicity to green algae, with EC_50_ values ranging from 0.032 mg/L to 0.374 mg/L. In contrast, chronic toxicity assessments revealed certain decomposed products to be more toxic than TFA itself. Specifically, TP2 (3.264 mg/L), TP3 (1.626 mg/L), TP4 (9.655 mg/L), and TP5 (8.690 mg/L) were harmful to fish. Moreover, TP2 (8.458 mg/L) and TP3 (5.496 mg/L) posed risks to daphnia. Additionally, TP1 (0.102 mg/L) exhibited toxicity to green algae, while TP2 (0.087 mg/L), TP3 (0.009 mg/L), TP4 (0.090 mg/L), and TP5 (0.091 mg/L) showed high toxicity levels to green algae.

Nevertheless, since the accumulated/sustainable outcomes indicate potential high toxicity of both TFA and its breakdown products toward certain aquatic organisms, it is crucial to conduct further risk assessments. These assessments will ascertain whether these pollutants indeed pose a risk of inducing toxic effects in the natural water environment.

## 3. Materials and Methods

### 3.1. Chemicals and Reagents

The standard analytical TFA (chemical purity = 99.30%) and five of its TPs (TP1 (purity = 95.36%); TP2 (purity = 95.68%); TP3 (purity = 94.16%); TP4 (purity = 91.38%); and TP5 (purity = 95.40%)) were provided by Dongbu Farm Hannong Co., Ltd. (Seoul, Korea). Methanol and acetonitrile (HPLC grade, Merck, Darmstadt, Germany), as well as formic acid (HPLC grade, Sigma Company, St. Louis, MO, USA), were utilized. Filtration employed 0.22 µm PTFE membrane needle filters (Pall Company, Port Washington, NY, USA), and all experiments were conducted using Milli-Q ultrapure water. Chemicals such as acetone, ethyl acetate, dichloromethane, petroleum ether, sodium chloride (AR grade, Xilong Scientific Co., Ltd., Shantou, Guangdong, China), and anhydrous magnesium sulphate (AR grade, Sinopharm Chemical Reagents Co., Ltd., Shanghai, China) were employed. Additionally, chromatography materials like octadecylsilane (C18, 50 μm, 60 A), graphitized carbon black (GCB, 60 μm), propyl ethylene diamine (PSA, 40~63 μm, 60 A), and multi-walled carbon nanotubes (MWCNTs ≥ 95%, 3~12 μm, BET ≥ 250 m^2^/g) were procured from Shanghai Anpu Experimental Technology Co., Ltd., Shanghai, in China.

Standard stock solutions (1000 mg/L) of individual pesticide and transformation products (TP1, TP2, TP3, TP4, TP5) were dissolved in HPLC-grade acetonitrile. Mixed solutions were prepared by dissolving TFA and the five TPs each at a concentration of 100 mg/L in acetonitrile. Mixed calibration graphs of standard solutions (15, 30, 50, 100, 500, 1000, and 2000 μg/L) were generated by serial dilutions of the above stock solution (10 mg/L) using acetonitrile. Similarly, matrix-matched standard solutions of the same concentration ranges were prepared using blank sample extracts for each corresponding standard solution.

### 3.2. Sample Preparation

Based on the instructions, blank samples were collected from a local orchard to ensure no TFA was available in the soils. Surface soils were collected from Nanchang City (Anthrosols), Fuzhou City (Ferralsols), Ganzhou City (Lixisols), and Shangrao City (Gleysols). The soils were air-dried, gently ground, and passed through a 0.25 mm mesh sieve. The basic physicochemical properties of each soil were examined using standard soil testing methods [36], and soil types were classified based on the World Reference Base for Soil Resources (WRB) [37] (Table S7).

For extracting TFA and its five metabolite residues from soil samples, the following procedures were employed: 5 ± 0.1 g of the sample was placed in a 50 mL polypropylene centrifugate tube, which was vortexed for 2 min to ensure even herbicide distribution. Subsequently, 10 mL of acetonitrile acidified with an additional 1% formic acid was added and vortexed for 2 min using a XW-18DL vortex mixer from Qiwei Instrument Co., Ltd., Hangzhou, China. Following this, 1.0 g of NaCl and 2.0 g of MgSO_4_ were introduced to the tube, vortexed for 1 min until the aqueous phase became clear, and centrifuged at 9000 rpm for 2 min using a Digicen 21 R desktop refrigerated centrifuge (Wiggens, Germany). The resulting upper organic phase (1.5 mL) was transferred to a 2 mL centrifuge tube with 5 mg of MWCNTs and 150 mg of MgSO_4_ for purification. After vortexing for 1 min, the supernatant underwent centrifugation at 6000 rpm for 5 min. The cleared supernatant was filtered through a 0.22 µm organic filter membrane and analyzed using UHPLC-QTOF-MS/MS.

### 3.3. Instrumental Parameters

All sample extracts underwent analysis using a UHPLC system (LC-20AD XR, Shimadzu Corporation, Kyoto, Japan) coupled with a SCIEX X500R Q-TOF-MS/MS instrument (AB SCIEX Corporation, Los Angeles, CA, USA) featuring a Turbo VTM source. The Waters Thermo scientific Hypersli Gold C8 column (2.1 × 100 mm, 1.7 µm) separated the compounds. The column was maintained at 40 °C to decrease the sample’s viscosity, while the autosampler was kept at 4 °C. A mixture of water (containing 0.1% *v*/*v* formic acid) (A) and acetonitrile (B) was used as the mobile phase at a flow rate of 0.35 mL/min. The gradient procedure was performed in the following steps: 0 min, 30% A; 1 min, 60% A; 1.5 min, 90% A; 2 min, 60% A; 2.5 min, 30% A. The total run time was 2.5 min, and all compounds were successfully separated, as shown in Figure 1. The retention times for TFA, TP1, TP2, TP3, TP4, and TP5 were 1.84, 2.25, 1.75, 1.39, 1.52 and 1.18 min with an injection volume of 4 μL.

Quantitative analysis of TFA and its five metabolites was conducted using a triple quadrupole mass spectrometer with an electrospray ionization source. The nebulizing gas pressure (nitrogen, 99.95% purity) was 15.0 psi, and the capillary voltage was set at 3500 V. The drying gas temperature was maintained at 320 °C, with a flow rate of 0.4 mL/min. Multiple reaction monitoring (MRM) was performed for 50 ms to detect TFA and its TPs. Two ion conversions were used for each compound, and collision tests were carried out to determine the most appropriate conversion. The collision energy and cone voltage were optimized in MRM mode to achieve the best sensitivity and resolution of each target compound. Analyte fragmentation voltages, collision energies, precursor ions and product ions are presented in Table 1.

### 3.4. Dissipation Experiments

We conducted TFA dissipation experiments in accordance with the NY/T 3150-2017 guidelines [25]. To maintain aerobic conditions, 20 g samples of agricultural soil from each type (Anthrosols, Ferralsols, Lixisols, Gleysols) were transferred to 250 mL flasks in triplicate. Subsequently, ultra-pure water was added during incubation to ensure the soil water content remained at 60% of its maximum water-holding capacity. Following this, 400 µL of a 100 mg/L working solution of TFA was added to each soil sample, resulting in an initial concentration of 2 mg/kg. The samples were then placed in a dark incubator at 25 ± 1 °C. Three parallel sub-samples were collected after treatments at 0, 1, 2, 4, 6, 12, 24, 48, and 96 h. The content of TFA and its five metabolites at each sampling time was determined using UHPLC-QTOF-MS/MS. Importantly, the water levels of the 250 mL flasks were regularly adjusted to maintain their initial water-holding capacity throughout the experiment.

For the sterilized conditions, a set of dissipation experiments was performed under sterile conditions. Sterilized soils (20 g each) were weighed and introduced into 250 mL flasks in triplicate. Notably, sterile water was added during the cultivation process to maintain the soil water content at 60% of the maximum water-holding capacity. Sub-samples were obtained as aerobic treatments, and UHPLC-QTOF-MS/MS determined the content of TFA and its five metabolites on 0, 1, 2, 6, 12, 24, 48, 96, and 192 h.

Under anaerobic conditions, soil samples were cultured for one month with an additive water layer with a 2 cm thickness. Gaseous N_2_ was consistently added to the incubation system to maintain stability. The soil samples were incubated in the dark at 25 ± 1 °C. Sub-samples were obtained as aerobic treatments, and UHPLC-QTOF-MS/MS determined the content of TFA and its five metabolites on days 0, 1, 2, 4, 6, 10, 30, 60, 90, and 120.

### 3.5. Theoretical Calculation of TFA Dissipation Reactions

The vibrational frequency and optimized geometries were inferred at B3LYP/6-311g (d) level using the Gaussian 16 Revision A.03 program [38]. Using the optimized structure, we performed single-point energy calculations at the B3LYP/6-311++ g (d, p) level. We applied a density-based SMD implicit solvation model analysis to the acetonitrile solution according to the following equation [39]. We confirmed all transition states by obtaining a negative value of the vibrational frequency, indicating a virtual frequency with only one imaginary frequency. Moreover, the reactive sites were evaluated by the Hirshfeld charges. In addition, the rapid advancement of computational chemistry enables the prediction of reactive sites in relatively large molecules, such as TFA.

### 3.6. Toxicity Prediction by ECOSAR

The toxicity of TFA and its five metabolites (TP1, TP2, TP3, TP4, and TP5) was evaluated using ECOSAR v 2.2 (www.epa.gov/tsca-screening-tools/ecological-structure-activity-relationships-ecosar-predictive-model) by inputting their molecular structures (.mol) into the program to determine the potential harm to fish, daphnia, and green algae.

### 3.7. Actual Field Samples

From July to August 2022, a field experiment was carried out in citrus-growing areas of Ganzhou (Lixisols) and Shangrao (Gleysols) in Jiangxi Province, China. Each site utilized a 667 m^2^ citrus orchard with 10-year-old trees averaging 10 m^2^ per tree. No prior TFA application or weed control was undertaken. The experiment followed the ‘Standard Operating Procedures for Field Efficacy Testing of Pesticide Residues’ and the updated ‘Guidelines for the Testing of Pesticide Residues in Crops: NY/T 788-2018’ by the Ministry of Agriculture [40]. We treated weeds in the plots with 70% TFA aqueous dispersion granules at 240.75 g a.i./hm^2^ [19]. Samples of Lixisols and Gleysols, each weighing at least 2 kg, were collected at different intervals following TFA application (at 2 h, 1, 3, 7, 14, 28 days) from five to ten random points within each plot. The samples were stored at −20 °C before analysis.

### 3.8. Date Processing

#### 3.8.1. Dissipation Kinetics Study

The dissipation kinetics of TFA in soil samples were simulated using first- (Equation (1)) and second-order (Equation (2)) kinetic equations [41]:

(1) $$C_{t}=C_{0}e^{-k_{1}t}$$

(2) $$\frac{1}{C_{t}}=\frac{1}{C_{0}}+k_{2}t$$

where C_t_ (mg/kg) and C_0_ (mg/kg) are the concentrations of TFA in the soil at incubation times t (d) and 0 (d), respectively, the values of “k_1_” and “k_2_” represent the rate constant of the first- (d^−1^) and second-order (kg/(mg·d)) kinetics, respectively.

The half-life (t_1/2_) was calculated according to Equation (3) [42]:

t_1/2_ = 0.693/k (3)

The rate of dissipation can be classified into four categories: easily degradable with a half-life of 30 or less, moderately degradable with a half-life between 30 and 90, lightly degradable with a half-life between 90 and 180, and poorly degradable with a half-life exceeding 180 [43].

#### 3.8.2. Method Validation

The LOQ, accuracy, precision, matrix effects (MEs), specificity, linearity, and stability of our analytical method were evaluated under European Union Guidance Document SANTE/11312/2021 [17].

A recovery study was conducted using five replicate spiked samples at three varying concentration levels (0.10, 0.50, and 2.00 mg/kg) for all target compounds to evaluate the precision and accuracy. Precision was reported as RSD% and measured as intra-day and inter-day reproducibility. Meanwhile, accuracy was expressed as the recovery (%). To differentiate between matrix-induced signal suppression/enhancement and extraction efficiency, ME were determined using Equation (4) [44]:

(4) $$ME=\left(\frac{K_{sam}-K_{std}}{K_{std}}\right)\times 100\%$$

K_sam_ and K_std_ are the standard curve slope in acetonitrile and matrix solution, respectively.

Sources of uncertainty were identified using the Fishbone diagram. The sample weight, reference materials (purity of the standard and preparation of the working standard), linear calibration curve interpolation, instrumental factors, matrix (recovery), and final volume were considered sources of uncertainty. Each uncertainty factor was calculated according to type A (standard deviation of the series of measured values) and type B (calibration certificate of the balance and pipette) uncertainties. The combined standard uncertainty (u_c_(y)) was calculated as the positive square root of the total variance obtained by combining all uncertainty components based on Equation (5). The expanded measurement uncertainty (U(y)) was calculated using a standard coverage factor (k) of 2 for an approximate level of confidence of 95% based on Equation (6) [45].

(5) $$u_{c}^{2}\left(y\right)=\overset{N}{\underset{i=1}{\sum }}{\left(\frac{\partial y}{\partial x_{i}}\right)}^{2}u^{2}\left(x_{i}\right)$$

(6) $$U\left(y\right)=k\left(y\right)$$

U(y) is the expanded uncertainty; k is the coverage factor (k) at a 95% confidence level; and u_c_(y) is the combined uncertainty.

We fitted data using OriginPro 2022 (OriginLab Corp., Northampton, MA, USA) with both first- and second-order dissipation models. Structural visualization was performed using Gauss View 6.0 software. Energy curves for all reactions were created using Indraw V6.0.4 software. Reported values are means from three independent replicates. Statistical differences were assessed using Duncan’s multiple range test, while Pearson correlation analysis was conducted using SPSS Statistics 22.0 (IBM SPSS, Somers, NY, USA) and GraphPad Prism 9.4.1 (GraphPad Software, San Diego, CA, USA) to scrutinize relationships among the means.

## 4. Conclusions

In summary, a rapid and precise method was developed using improved QuEChERS pre-treatment and UHPLC-QTOF-MS/MS technology to detect TFA and its transformation products (TP1, TP2, TP3, TP4, and TP5) in soil, and its performance was compared with published data on TFA and its metabolites. Taken together, this method offers sufficient advantages such as lower limits of quantification, lower relative standard deviation values, and a wider linear range.

Furthermore, this study described the dissipation kinetics (including half-life) of TFA in four different types of soil. By continuously monitoring the concentrations of TFA and its five transformation products in various soils, we determined the major transformation products under different conditions. TFA dissipation under aerobic conditions followed first-order kinetics. In contrast, its dissipation under sterilized and anaerobic conditions fitted second-order kinetics. Additionally, we predicted that TFA could be transformed into TP1 and TP2 and assumed that the toxicity of the five transformation products was higher to green algae than that of the parent TFA. Respectively, our study provides a scientific basis for the environmental fate and risk assessment of TFA and its homologous pyrimidinedione herbicides.

## Figures and Tables

**Table 1:** Mass-spectrometry parameters to determine TFA and its TPs.

Compound	Molecular Formula	Retention Time (min)	Fragmentor (V)	Precursor Ion (*m*/*z*)	Qualifier Ion (*m*/*z*)	Q/q *	Collision Energy (eV)
TFA	C_19_H_18_ClF_4_N_3_O_5_S	1.84	120	512.1	480.0	Q	37
					426.0	q	
TP1	C_18_H_16_ClF_4_N_3_O_5_S	2.25	120	498.1	480.0	Q	35
					381.0	q	
TP2	C_15_H_11_ClF_4_N_2_O_4_S	1.75	100	427.2	409.0	Q	23
					355.0	q	
TP3	C_15_H_12_ClF_4_N_3_O_3_S	1.39	140	426.1	409.0	Q	27
					381.0	q	
TP4	C_15_H_11_ClF_4_N_2_O_5_S	1.51	100	443.1	368.8	Q	51
					352.9	q	
TP5	C_15_H_13_ClF_4_N_2_O_5_S	1.18	140	445.1	370.9	Q	33
					354.9	q	

* Q represents quantification ion transition and q represents confirmation ion transition.

**Table 2:** Calibration equations, R^2^, matrix effect, and LOQ in detecting TFA and its TPs.

Compound	Matrix	Regression Equation	R^2^	LOQ	Matrix Effect
				(µg/kg)	(%)
TFA	Acetonitrile	y = 6269.8x + 102031	0.9999	-	-
	Anthrosols	y = 7291.8x − 257895	0.9954	2.00	0.82
	Ferralsols	y = 6958.8x + 37158	0.9987	2.00	1.10
	Lixisols	y = 8488.6x − 3944.7	0.9988	2.00	0.84
	Gleysols	y = 7537.7x − 162161	0.9986	2.00	1.05
TP1	Aceronitrile	y = 5544x + 105261	0.9995	-	-
	Anthrosols	y = 6232.2x − 203978	0.9973	2.00	0.97
	Ferralsols	y = 5900.7x + 23821	0.9992	2.00	0.92
	Lixisols	y = 7229.6x + 2410.5	0.9983	2.00	1.08
	Gleysols	y = 7509.2x − 175689	0.9975	2.00	0.90
TP2	Aceronitrile	y = 6408.3x + 26722	0.9997	-	-
	Anthrosols	y = 5325.9x − 158761	0.9985	2.00	1.07
	Ferralsols	y = 4868.1x + 42083	0.9987	2.00	0.84
	Lixisols	y = 6173.6x + 16669	0.9968	2.00	1.12
	Gleysols	y = 6411.3x − 134715	0.9988	2.00	1.16
TP3	Aceronitrile	y = 5789.8x − 6729.1	0.9974	-	-
	Anthrosols	y = 4791.6x − 113832	0.9992	2.00	1.17
	Ferralsols	y = 4365.3x + 60164	0.9963	2.00	0.80
	Lixisols	y = 5527.8x + 1069	0.9984	2.00	1.06
	Gleysols	y = 5741.7x − 135177	0.9973	2.00	0.98
TP4	Aceronitrile	y = 6306x − 64930	0.9971	-	-
	Anthrosols	y = 4548.5x − 117272	0.9989	2.00	1.19
	Ferralsols	y = 4103.4x + 55452	0.9963	2.00	0.75
	Lixisols	y = 5181.8x − 6860.6	0.9991	2.00	1.10
	Gleysols	y = 5367x − 142507	0.9949	2.00	0.94
TP5	Aceronitrile	y = 4551.5x + 60842	0.9988	-	-
	Anthrosols	y = 5048x − 149091	0.9978	2.00	0.69
	Ferralsols	y = 4760.9x + 41728	0.9975	2.00	0.81
	Lixisols	y = 5567.8x + 47773	0.9970	2.00	1.03
	Gleysols	y = 6091.3x − 137091	0.9980	2.00	1.13

**Table 3:** Comparison of the method performance for TFA and its TPs.

Compounds	Instrument Used	LOQ (µg/kg)	LR (µg/kg)	ME%	RSD%	References
TFA	HPLC-MS	(8.80~10.30) × 10^3^	0.01~1.0 *	−8.64~962	0.25~9.31	[19]
TFA	UHPLC-MS/MS	10	5~1000	0.58~0.97	1.00~11.40	[11]
TFA	UHPLC-QTOF-MS/MS	2	15~2000	0.82~1.10	0.46~12.93	Present work
TP1	UHPLC-MS/MS	10	5~1000	0.61~0.89	1.30~7.60	[11]
TP1	UHPLC-QTOF-MS/MS	2	15~2000	0.92~1.08	0.28~8.87	Present work
TP2	UHPLC-QTOF-MS/MS	2	15~2000	0.84~1.16	2.99~10.75	Present work
TP3	UHPLC-QTOF-MS/MS	2	15~2000	0.80~1.17	3.95~9.97	Present work
TP4	UHPLC-MS/MS	10	5~1000	0.65~0.92	1.80~9.90	[11]
TP4	UHPLC-QTOF-MS/MS	2	15~2000	0.75~1.19	1.61~11.55	Present work
TP5	UHPLC-MS/MS	10	5~1000	0.64~0.95	1.10~9.40	[11]
TP5	UHPLC-QTOF-MS/MS	2	15~2000	0.69~1.13	1.49~10.38	Present work

Limit of quantification (LOQ), linear range (LR), matrix effects (ME), relative standard deviation (RSD). * mg/L.

## Data Availability

Data are contained within the article and Supplementary Materials.

## Supplementary Materials

The following supporting information can be downloaded at: https://www.mdpi.com/article/10.3390/ijms25158367/s1.
