# Supplementary material for: Concurrent Analysis of Tiafenacil and Its Transformation Products in Soil by Using Newly Developed UHPLC-QTOF-MS/MS-Based Approaches
Source: Int J Mol Sci. 2024 Jul 31;25(15):8367. doi: 10.3390/ijms25158367 (PMC11313644; doi:10.3390/ijms25158367)
Supplement: Supplementary file 1 [file ijms-25-08367-s001.zip › ijms-3118433-supplementary.pdf]

Supplementary material for Concurrent Analysis of Tiafenacil and Its Transformation Products in Soil by Using Newly Developed UHPLC-QTOF-MS/MS-Based Approaches

Wenwen Zhou [1,*,†], Anqi Yan [2,†], Shujie Zhang [1], Dayong Peng [3] and Jun Li [1,*]

[1] College of Food Science and Engineering, Jiangxi Agricultural University, Nanchang 330045, China; zhangsj@163.com
[2] School of Agriculture, Food and Ecosystem Sciences, University of Melbourne, Parkville VIC 3052, Australia; anqi.yan1@student.unimelb.edu.au
[3] College of Chemistry and Materials, Jiangxi Agricultural University, Nanchang 330045, China; dayongpeng@163.com
[*] Correspondence: wenwenzhou@jxau.edu.cn (W.Z.); lijunbio@jxau.edu.cn (J.L.); Tel.: +86-13517000002 (W.Z.)
[†] These authors contributed equally to this work.

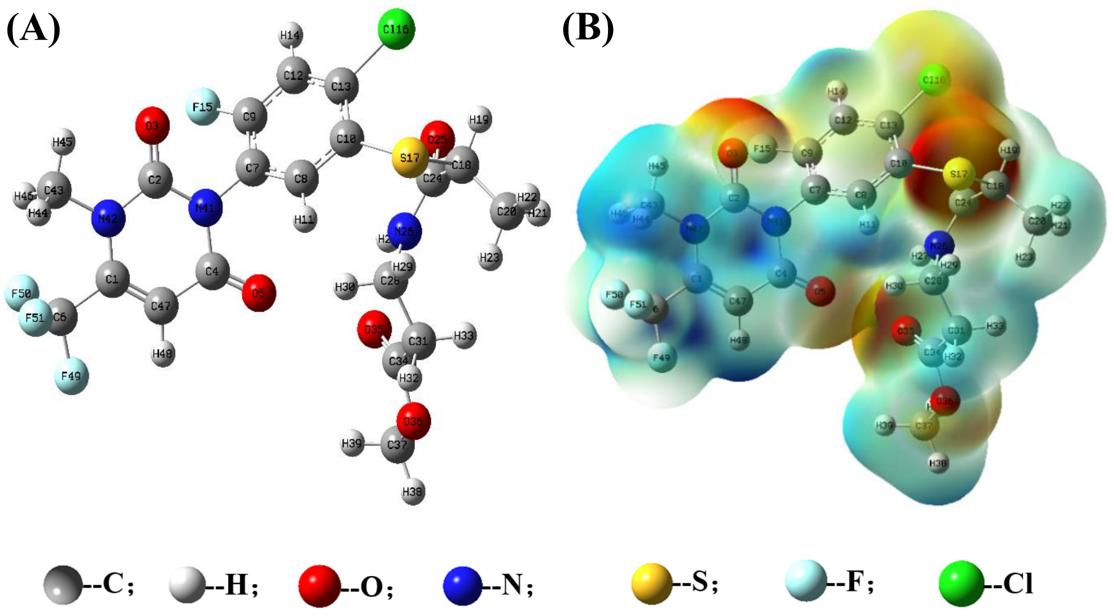

Figure S1. The molecular structure and molecular electrostatic potential of TFA. (A) TFA molecular structure. (B) Molecular electrostatic potential (MEP), and the red and blue regions corresponded to most negative and positive MEP values, respectively).

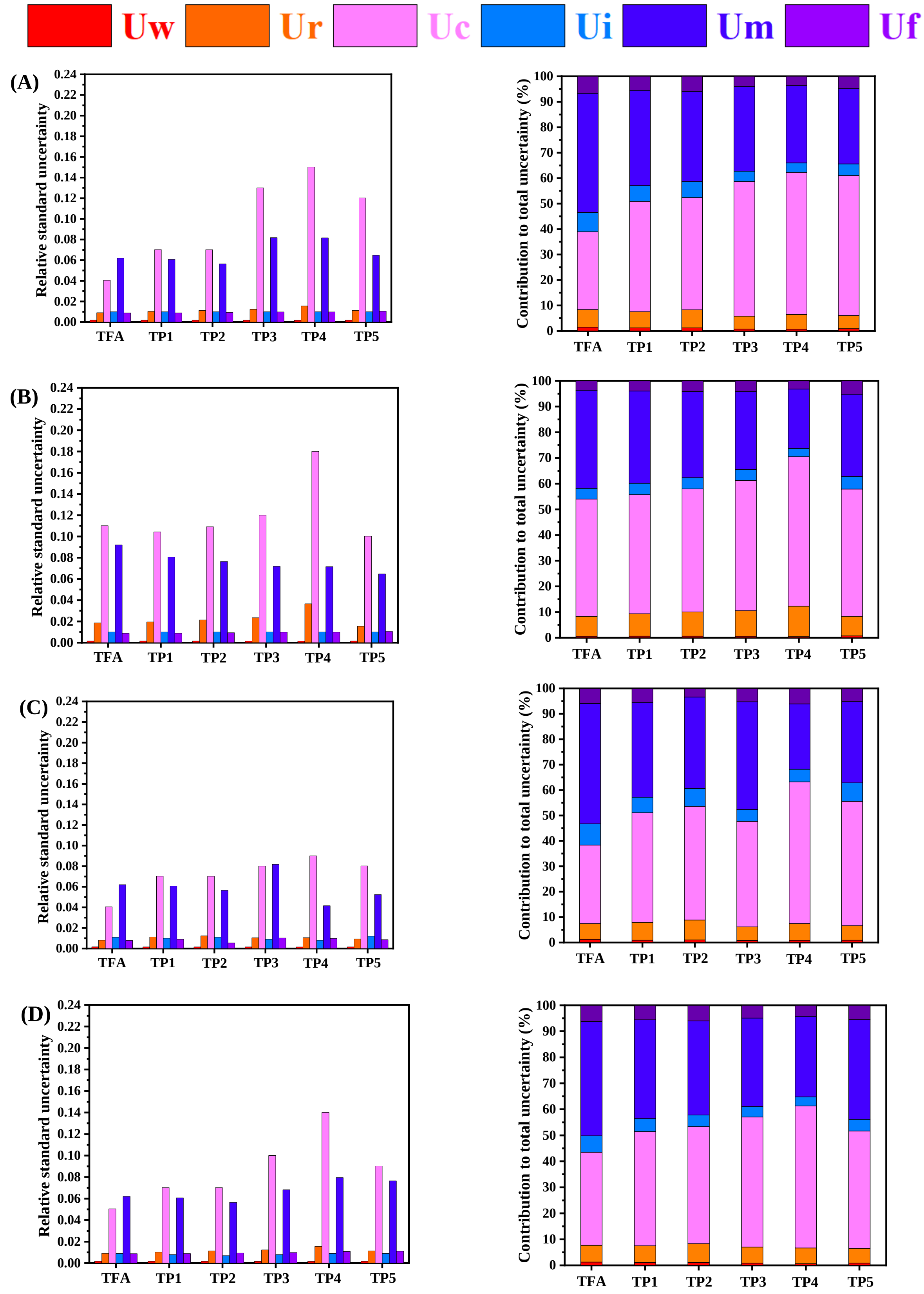

Figure S2. Relative uncertainty (left) and contribution of each parameter of uncertainty (right) in **(A)** Anthrosols, **(B)** Ferralsols, **(C)** Lixisols and **(D)** Gleysols (Uw: sample weight, Ur: reference material, Uc: calibration curve, Ui: instrument, Um: recovery, Uf: final volume).

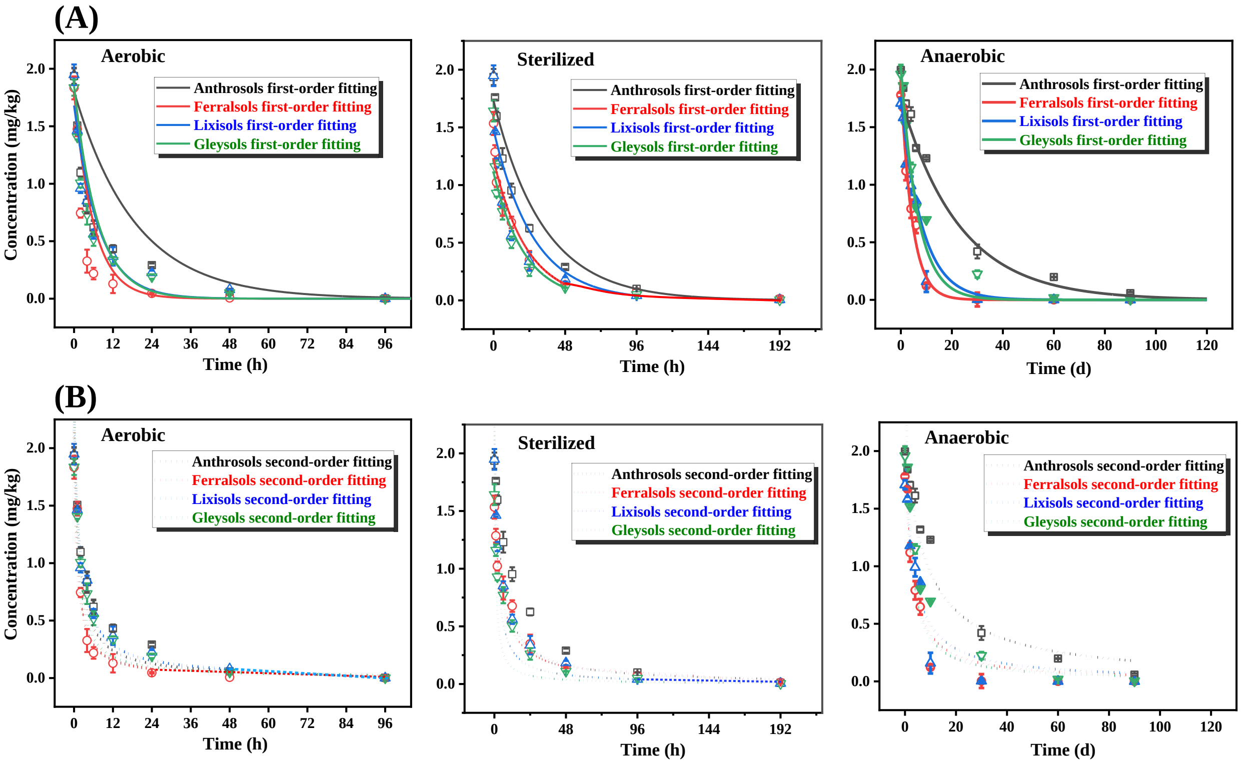

Figure S3. TFA dissipation dyanmics and kinetics in different soils: **(A)** the first-order fitting and **(B)** the second-order fitting.

Table S1. Accuracy, precision, and measurement uncertainty of the method for detecting TFA and TPs in the four soil types.

Compound	Matrix	Spiked level (mg /kg)	Recoveries (%)					Average recoveries (%)	RSDr (%)	RSDR (%)	Uncertainty /Result (%)
TFA	Anthrosols	0.1	87.5	89.9	85.3	89.1	88.2	88.0	2.0	3.2	22.0
		0.5	87.4	92.3	88.4	90.9	88.9	89.6	2.2	3.5	20.4
		2.0	89.3	98.0	100.0	96.1	96.4	95.9	4.2	6.9	14.1
	Ferralsols	0.1	80.9	82.2	94.3	76.3	98.0	86.3	10.8	12.9	23.7
		0.5	85.7	86.2	88.1	87.6	88.5	87.2	1.4	2.1	22.8
		2.0	93.0	93.3	94.1	93.2	93.5	93.4	0.5	0.6	16.6
	Lixisols	0.1	89.5	84.0	91.7	87.6	87.8	86.4	4.9	6.0	23.7
		0.5	91.4	87.8	87.8	86.2	86.1	87.0	1.0	1.1	23.0
		2.0	88.7	95.0	94.3	94.5	94.5	94.8	0.7	1.0	15.2
	Gleysols	0.1	97.1	100.1	104.9	93.4	104.3	100.0	4.8	5.9	10.1
		0.5	99.1	104.7	105.3	99.3	100.0	101.7	3.0	3.9	8.3
		2.0	97.4	101.2	103.3	102.4	103.7	101.6	2.5	3.1	8.4
TP1	Anthrosols	0.1	80.6	89.2	89.1	89.6	89.1	89.3	0.3	0.3	20.7
		0.5	87.0	93.6	93.7	94.2	93.3	93.3	1.2	1.8	16.8
		2.0	95.9	101.9	95.0	100.3	101.7	97.5	5.8	6.6	12.5
	Ferralsols	0.1	108.1	97.9	87.7	100.7	101.5	99.2	7.5	4.8	10.8
		0.5	109.3	94.4	90.0	97.2	102.8	98.7	7.6	8.8	11.3
		2.0	109.0	97.7	91.1	89.3	104.0	98.2	8.5	6.5	6.8
	Lixisols	0.1	101.6	109.8	89.2	93.3	95.6	97.9	8.2	6.1	12.1
		0.5	86.8	100.6	92.2	90.2	107.1	95.4	8.7	8.9	14.6
		2.0	103.2	106.0	88.7	101.0	105.3	100.8	7.0	7.1	9.2
	Gleysols	0.1	85.7	105.3	101.4	93.1	98.1	96.7	7.9	4.8	13.3
		0.5	108.9	99.9	95.1	94.0	105.3	100.6	6.4	3.6	9.4
		2.0	100.9	100.9	91.9	100.5	92.0	97.2	5.0	6.9	7.8
TP2	Anthrosols	0.1	93.0	98.4	92.8	91.2	92.4	93.6	3.0	5.8	16.4
		0.5	93.3	98.7	102.6	98.5	91.5	96.9	4.6	6.5	13.1
		2.0	104.9	91.1	90.9	108.3	87.5	96.5	9.7	3.5	8.5
	Ferralsols	0.1	86.8	87.1	92.0	108.1	97.4	94.3	9.4	6.7	15.7
		0.5	96.3	93.7	99.1	93.4	101.7	96.8	3.7	6.5	13.2
		2.0	105.4	95.9	102.9	92.8	88.2	97.0	7.3	9.2	11.0
	Lixisols	0.1	92.0	95.3	109.0	85.8	109.6	98.3	10.8	8.1	11.7
		0.5	86.3	99.9	92.4	90.4	110.0	95.8	9.8	3.1	14.2
		2.0	87.2	102.5	88.1	95.0	103.9	95.3	8.2	6.8	9.7
	Gleysols	0.1	109.1	102.8	87.6	100.0	98.6	99.6	7.9	6.5	10.4
		0.5	96.6	107.4	89.7	101.0	91.5	97.2	7.4	8.0	12.8
		2.0	106.4	87.4	92.2	97.7	100.0	96.7	7.5	6.2	10.3
TP3	Anthrosols	0.1	96.1	98.9	97.7	109.6	88.7	98.2	7.6	6.4	11.8
		0.5	105.0	108.0	108.4	99.1	109.4	106.0	4.0	9.5	14.0

		2.0	98.7	110.0	109.5	109.1	93.9	104.2	7.2	8.9	9.8
		0.1	86.9	86.6	96.0	98.7	94.2	92.5	5.9	7.5	27.5
	Ferralsols	0.5	105.6	94.2	85.4	107.4	108.1	100.1	10.0	8.4	19.9
		2.0	89.0	106.2	94.0	94.0	105.4	97.7	7.8	6.0	22.3
		0.1	105.5	105.5	102.1	104.2	92.0	101.9	5.6	4.9	18.1
	Lixisols	0.5	94.7	107.2	102.5	109.6	109.3	104.7	6.0	5.6	15.3
		2.0	95.2	98.4	106.4	93.8	98.3	98.4	5.0	7.3	11.6
		0.1	99.9	89.3	107.4	101.0	88.8	97.3	8.3	7.8	22.7
	Gleysols	0.5	109.9	107.1	98.9	86.8	104.3	101.4	9.0	8.4	18.6
		2.0	91.6	98.3	85.2	92.4	89.4	91.4	5.2	6.2	16.6
TP4		0.1	100.9	91.5	102.2	107.7	89.8	98.4	7.7	8.3	31.6
	Anthrosols	0.5	91.4	98.3	94.4	85.6	104.0	94.7	7.3	8.2	35.3
		2.0	102.4	106.6	104.4	92.9	88.8	99.0	7.8	7.4	21.0
		0.1	100.5	88.4	97.0	86.9	94.6	93.5	6.2	8.1	36.5
	Ferralsols	0.5	92.0	94.6	104.2	99.5	99.2	97.9	4.8	8.9	32.1
		2.0	92.7	88.5	109.5	95.7	92.8	95.8	8.4	7.0	14.2
		0.1	86.9	85.3	98.3	89.3	96.4	91.2	6.4	8.6	38.8
	Lixisols	0.5	96.3	92.6	93.3	95.4	94.0	94.3	1.6	7.6	35.7
		2.0	85.3	101.6	90.5	86.4	95.7	91.9	7.4	4.5	18.1
		0.1	106.3	88.8	102.9	103.5	104.4	101.2	7.0	1.7	28.8
	Gleysols	0.5	92.1	104.7	89.8	106.3	106.8	99.9	8.3	3.6	30.1
		2.0	85.2	93.9	109.9	107.2	88.0	96.8	11.6	7.5	23.2
		0.1	86.5	85.7	92.0	103.3	106.3	105.3	10.1	6.7	7.7
	Anthrosols	0.5	88.9	89.4	92.5	96.8	95.5	89.5	3.8	1.7	20.5
		2.0	104.9	101.6	97.4	85.6	91.8	97.2	8.0	3.2	12.8
		0.1	100.9	103.4	89.3	94.3	98.0	96.4	5.7	9.8	10.6
	Ferralsols	0.5	95.3	92.0	90.4	108.1	93.3	102.8	7.4	5.1	17.2
		2.0	97.8	97.4	101.5	108.8	97.2	92.2	4.9	1.5	12.8
TP5		0.1	90.8	91.8	92.1	101.1	109.7	109.3	8.4	2.3	8.7
	Lixisols	0.5	91.1	92.3	97.2	98.4	99.4	111.1	3.9	7.8	19.0
		2.0	98.2	101.1	98.2	100.3	105.2	89.7	2.9	5.6	10.3
		0.1	109.6	101.0	106.2	101.5	99.1	86.4	4.2	1.6	23.6
	Gleysols	0.5	89.0	108.9	88.0	102.2	87.3	103.3	10.4	7.6	6.7
		2.0	104.4	86.7	105.3	95.9	98.1	105.2	7.7	6.9	4.8

Average recoveries, RSDr (%) and RSDR (%) for target compounds from different matrices at three spiked levels. RSDr: Intra-day is the relative standard deviation for repeatability (n=5); RSDR: Inter-day is the relative standard deviation for reproducibility (n=5).

* Expand uncertainty (k=2, confidence level=95%).

Table S2. Degradation kinetic models and related characteristics of TFA in soil under different conditions.

Model	Soil type	Aerobic			Sterilized			Anaerobic			
		kinetic model	$t_{1/2}$ (h)	R^2	kinetic model	$t_{1/2}$ (h)	R^2	kinetic model	$t_{1/2}$ (d)	$t_{1/2}$ (h)	R^2
First-order kinetic	Anthrosols	Ct=1.8021e-0.0533t	13.00	0.8084	Ct=1.7310e-0.0298t	23.26	0.9864	Ct=1.7513e-0.0436t	15.89	381.36	0.9864
	Ferralsols	Ct=1.7893e-0.1668t	4.15	0.9582	Ct=1.2173e-0.0449t	15.43	0.9777	Ct=1.9113e-0.2275t	3.05	73.20	0.9758
	Lixisols	Ct=1.6803e-0.1314t	5.27	0.9393	Ct=1.4786e-0.0375t	18.48	0.9899	Ct=1.7618e-0.1206t	5.75	138.00	0.9882
	Gleysols	Ct=1.8493e-0.1387t	5.00	0.8486	Ct=1.1065e-0.0482t	14.38	0.9859	Ct=2.0223e-0.1507t	4.60	110.40	0.9873
Second-order kinetic	Anthrosols	1/Ct=1/2.4675+0.0652t	10.63	0.9604	1/Ct=1/2.2530+0.0371t	18.68	0.8900	1/Ct=1/2.0605+0.0572t	12.12	290.88	0.9707
	Ferralsols	1/Ct=1/2.1383+0.1708t	4.06	0.9131	1/Ct=1/1.4890+0.0586t	11.83	0.9433	1/Ct=1/2.0493+0.1967t	3.52	84.48	0.9494
	Lixisols	1/Ct=1/2.3019+0.2049t	3.38	0.9972	1/Ct=1/2.3041+0.0517t	13.40	0.9891	1/Ct=1/1.9354+0.1513t	4.58	109.92	0.9528
	Gleysols	1/Ct=1/2.4618+0.1866t	3.71	0.8926	1/Ct=1/1.5736+0.0606t	11.44	0.9715	1/Ct=1/3.5252+0.2400t	2.89	69.36	0.8703

Table S3. The relationship between the half-life of TFA in soil and its underlying physicochemical properties examined under aerobic, sterilized, and anaerobic conditions.

	Aerobic	pH	OM	CEC	Clay	TON
Aerobic	1.000	-0.778	-0.864	0.965	0.347	0.083
pH	-0.778	1.000	0.921	-0.879	-0.806	-0.432
OM	-0.864	0.921	1.000	-0.932	-0.722	-0.113
CEC	0.965	-0.879	-0.932	1.000	0.481	0.167
Clay	0.347	-0.806	-0.722	0.481	1.000	0.370
TON	0.083	-0.432	-0.113	0.167	0.370	1.000
	Anaerobic	pH	OM	CEC	Clay	TON
Anaerobic	1.000	-0.803	-0.863	0.980	0.341	0.146
pH	-0.803	1.000	0.921	-0.879	-0.806	-0.432
OM	-0.863	0.921	1.000	-0.932	-0.722	-0.113
CEC	0.980	-0.879	-0.932	1.000	0.481	0.167
Clay	0.341	-0.806	-0.722	0.481	1.000	0.370
TON	0.146	-0.432	-0.113	0.167	0.370	1.000
	Sterilized	pH	OM	CEC	Clay	TON
Sterilized	1.000	-0.741	-0.711	0.846	0.298	0.190
pH	-0.741	1.000	0.921	-0.879	-0.806	-0.432
OM	-0.711	0.921	1.000	-0.932	-0.722	-0.113
CEC	0.846	-0.879	-0.932	1.000	0.481	0.167
Clay	0.298	-0.806	-0.722	0.481	1.000	0.370
TON	0.190	-0.432	-0.113	0.167	0.370	1.000

OM: Organic matter content, CEC: Cation exchange capacity, TON: Total organic nitrogen.

Table S4. Natural charge distribution of TFA at the level of DFT/B3LYP/6-311G(d).

Atom	No.	Charge distribution	Atom	No.	Charge distribution
C	1	0.286	H	27	0.337
C	2	0.965	C	28	-0.268
O	3	-0.480	H	29	0.234
C	4	0.717	H	30	0.277
O	5	-0.486	C	31	-0.480
C	6	0.838	H	32	0.229
C	7	0.250	H	33	0.240
C	8	-0.111	C	34	0.700
C	9	0.343	O	35	-0.494
C	10	-0.361	O	36	-0.508
H	11	0.229	C	37	-0.345
C	12	-0.158	H	38	0.213
C	13	-0.256	H	39	0.217
H	14	0.249	H	40	0.218
F	15	-0.280	N	41	-0.877
Cl	16	0.153	N	42	-0.805
S	17	0.385	C	43	-0.430
C	18	-0.543	H	44	0.235
H	19	0.284	H	45	0.261
C	20	-0.556	H	46	0.235
H	21	0.218	C	47	-0.260
H	22	0.212	H	48	0.240
H	23	0.212	F	49	-0.259
C	24	0.691	F	50	-0.256
O	25	-0.497	F	51	-0.259
N	26	-0.703			

Table S5. The bond order and bond length of TFA at the level of DFT/B3LYP/6-311G(d).

Chemical bond	Bond length	Bond order	Chemical bond	Bond length	Bond order
C(1)-C(6)	1.518	1.0	C(18)-C(20)	1.533	1.0
C(1)-N(42)	1.378	1.0	C(18)-C(24)	1.533	1.0
C(1)-C(47)	1.350	2.0	C(20)-H(21)	1.096	1.0
C(2)-O(3)	1.215	2.0	C(20)-H(22)	1.095	1.0
C(2)-N(41)	1.402	1.0	C(20)-H(23)	1.091	1.0
C(2)-N(42)	1.408	1.0	C(24)-O(25)	1.246	2.0
C(4)-O(5)	1.221	2.0	C(24)-N(26)	1.357	1.5
C(4)-N(41)	1.419	1.0	N(26)-H(27)	1.020	1.0
C(4)-N(47)	1.448	1.0	N(26)-C(28)	1.470	1.0
C(6)-F(49)	1.339	1.0	C(28)-H(29)	1.087	1.0
C(6)-F(50)	1.349	1.0	C(28)-H(30)	1.091	1.0
C(6)-F(51)	1.351	1.0	C(28)-C(31)	1.536	1.0
C(7)-C(8)	1.389	1.5	C(31)-H(32)	1.094	1.0
C(7)-C(9)	1.396	1.5	C(31)-H(33)	1.097	1.0
C(7)-N(41)	1.430	1.0	C(34)-O(35)	1.231	2.0
C(8)-C(10)	1.401	1.5	C(34)-O(36)	1.373	1.0
C(8)-H(11)	1.082	1.0	O(36)-C(37)	1.780	1.0
C(9)-C(12)	1.386	2.0	C(37)-H(38)	1.088	1.0
C(9)-F(15)	1.339	1.0	C(37)-H(39)	1.092	1.0
C(10)-C(13)	1.405	1.5	C(37)-H(40)	1.092	1.0
C(10)-S(17)	1.785	1.0	N(42)-C(43)	1.490	1.0
C(12)-C(13)	1.396	1.5	C(43)-H(44)	1.089	1.0
C(12)-H(14)	1.083	1.0	C(43)-H(45)	1.087	1.0
C(13)-Cl(16)	1.744	1.0	C(43)-H(46)	1.089	1.0
S(17)-C(18)	1.860	1.0	C(47)-H(48)	1.075	1.0
C(18)-H(19)	1.090	1.0			

Table S6. Acute and chronic toxicity predictions for TFA and its TPs.

Compounds	Acute toxicity (mg/L)				Chronic toxicity (mg/L)		
	Log Kow	Fish (LC_{50})	Daphnia (LC_{50})	Green algae (EC_{50})	Fish	Daphnia	Green algae
TFA	1.378	352.000	905.000	571.000	19.600	101.000	156.000
TP1	1.069	2016.184	1451.282	0.374	14.482	46.803	0.102
TP2	2.106	455.992	113.899	0.320	3.264	8.458	0.087
TP3	0.858	226.186	201.973	0.032	1.626	5.496	0.009
TP4	1.293	1345.230	770.681	0.332	9.655	29.752	0.090
TP5	1.376	1211.150	636.045	0.334	8.690	26.297	0.091

Not harmful   Harmful   Toxic   Very toxic

Table S7. General physicochemical properties of the selected soils.

Soil type*	Location	Texture	pH	OM (%)	CEC (cmol/kg)	Clay (%)	TON （%）
Anthrosols	Nanchang	Loam	4.53	7.99	26.15	38.11	0.11
Ferralsols	Fuzhou	Sandy loam	6.05	15.06	12.19	40.35	0.12
Lixisols	Ganzhou	Sandy loam	7.12	19.95	11.99	29.80	0.13
Gleysols	Shangrao	Silt loam	8.02	18.33	11.00	29.32	0.08

*Soil classification according to the World Reference Base for Soil Resources.

OM: Organic matter content.

CEC: Cation exchange capacity

TON: Total organic nitrogen
